# Quality of Life in Men Treated for Androgenetic Alopecia via a Digital Healthcare Platform: A Retrospective Cohort Study

**DOI:** 10.1111/jocd.70999

**Published:** 2026-06-17

**Authors:** Angela C. Bersch‐Ferreira, Daniel Reisel, Ashley K. Clift, John C. T. Chao, Hans Johnson, David R. Huang, Hudson Dutra, Gabriel Guimarães

**Affiliations:** ^1^ Department of Clinical Research MANUAL LTDA Barueri São Paulo Brazil; ^2^ Department of Clinical Research A Beneficência Portuguesa de São Paulo São Paulo Brazil; ^3^ Department of Clinical Research Menwell Limited London UK; ^4^ EGA Institute for Women's Health University College London London UK; ^5^ Department of Surgery & Cancer Imperial College London London UK; ^6^ Department of Digital Health and Care, School of Engineering Mathematics and Technology University of Bristol Bristol UK; ^7^ Department of Dermatology Lusíada University Center Santos São Paulo Brazil

**Keywords:** androgenetic alopecia, hair loss, men's health, quality of life, telemedicine, treatment adherence

## Abstract

**Background:**

Androgenetic alopecia (AGA) is a common chronic condition significantly impacting quality of life (QoL), but few studies have explored treatment outcomes in real‐world digital health settings.

**Aims:**

The objective of this study is to evaluate the effects of AGA treatment delivered through a digital healthcare platform on patient‐reported QoL outcomes over 6 months, and identify factors associated with the magnitude of improvement.

**Patients/Methods:**

We undertook a before‐and‐after cohort study using prospectively collected data from men enrolled on a digital AGA service in Brazil. QoL was assessed using a 16‐item hair‐specific questionnaire at baseline and 6 months. Multivariable linear regression explored associations between QoL change and variables including baseline QoL score, age, adherence (defined as continuous prescription renewals within the programme), and medication potency.

**Results:**

The mean total QoL score improved from 89.07 to 76.78, yielding a mean change of −12.29 points (95% CI −14.44 to −10.14; *p* < 0.001; lower scores denote better QoL). Adherence was the strongest predictor of improvement: participants who maintained full engagement with the programme achieved a mean QoL gain roughly double that of those with interrupted engagement (*p* < 0.001). Lower baseline QoL and older age were also associated with greater improvements (*p* = 0.040), whereas medication potency was not.

**Conclusion:**

Treatment of AGA via a fully digital care model was associated with statistically significant improvements in hair‐loss‐related QoL over 6 months. The magnitude of improvement was greater among participants with sustained programme engagement. These findings support the potential value of digital care models for AGA, while recognizing that causal inference is limited by the observational design and absence of an in‐person comparator.

## 1 Introduction

Androgenetic alopecia (AGA) is a highly prevalent chronic condition that affects a substantial proportion of the male population worldwide [1, 2]. Characterized by progressive hair loss, AGA has a well‐documented psychosocial impact, including lower self‐esteem, reduced quality of life (QoL), and increased levels of anxiety and depression [3, 4].

Effective treatment options for AGA include pharmacological therapies such as topical minoxidil and oral 5‐alpha‐reductase inhibitors (e.g., finasteride or dutasteride), complemented by adjunctive therapies like low‐level light therapy, [5] nutraceuticals, [6] microneedling [7] and, in more advanced cases, hair transplantation [8, 9, 10]. Despite the availability of multiple evidence‐based treatments, achieving satisfactory clinical outcomes is often compromised by poor adherence. Pharmacological treatments for androgenetic alopecia typically require several months of consistent use to produce visible effects contributing to early discontinuation and patient frustration [9]. Treatment discontinuation leads to the progressive worsening of hair loss and the reversal of any previously achieved benefits [11, 12, 13].

While digital health platforms are increasingly used to support the management of chronic conditions, including AGA, real‐world evidence on patient‐reported outcomes within these models remains limited. Little is known about how quality of life evolves over time in patients undergoing treatment for AGA through digital care platforms. Understanding whether QoL improves during treatment—and how factors such as treatment adherence may influence this improvement—is essential for designing patient‐centerd care strategies in digital settings [14, 15]. These platforms have the potential to reduce logistical barriers, improve convenience, and foster higher levels of treatment continuity. However, real‐world data assessing whether improved adherence within digital care models translates into measurable benefits for patients—particularly in quality of life—are scarce.

This study aims to quantify changes in quality of life (QoL) among men undergoing treatment for androgenetic alopecia (AGA) within a digital healthcare platform by comparing QoL scores at baseline and at 6‐month follow‐up. Additionally, we explore whether treatment adherence is associated with greater improvements in QoL.

The delivery context of treatment may also influence outcomes. Digital healthcare platforms differ from conventional in‐person care in several respects that could plausibly affect treatment response and patient experience. They may improve adherence through automated reminders, asynchronous clinician support, simplified follow‐up pathways, and home delivery of medication, thereby addressing recognized barriers to long‐term treatment persistence. They may also provide support beyond prescribing alone, including education, progress tracking, and structured follow‐up, which may help lessen the psychological burden associated with hair loss. In the absence of a direct comparator group, this study does not compare digital and face‐to‐face care directly, but instead characterizes outcomes within this distinct model of care.

## 2 Methods

### 2.1 Study Design and Setting

This is a retrospective before‐and‐after analysis using prospectively collected data from a real‐world cohort of men enrolled at (https://www.manual.com.br/) a Brazilian digital healthcare platform. The platform provides medical consultations, prescription treatments, and follow‐up services for men with androgenetic alopecia. The study was designed and reported in accordance with the STROBE (Strengthening the Reporting of Observational Studies in Epidemiology) guidelines, and the completed STROBE checklist is provided as Supporting Information.

### 2.2 Participants and Eligibility Criteria

Men were eligible for inclusion if they were 18 years of age or older, had a clinical diagnosis of androgenetic alopecia confirmed by a physician affiliated with the digital healthcare platform, had received at least two prescriptions for pharmacological treatment for hair loss, such as minoxidil, finasteride, or dutasteride, through the platform, and had completed at least two quality‐of‐life (QoL) assessments, including one at baseline and one within the 6‐month follow‐up window. Exclusion criteria included individuals receiving only cosmetic treatments, such as shampoo and biotin, without pharmacological therapy, as well as those who initiated pharmacotherapy but later canceled or discontinued treatment before six months.

### 2.3 Data Sources and Collection

The digital healthcare platform Hair‐loss Programme is delivered entirely online. Prospective users begin by completing a detailed digital intake form covering medical history, pattern of hair loss, treatment goals and lifestyle considerations. A board‐certified physician reviews this information asynchronously and designs an individualized treatment plan based on internal clinical guidelines, typically combining oral and/or topical therapies. Once approved, the medications are dispensed and shipped directly to the patient's home in quarterly packages. Users retain full control over their subscription and can pause or cancel deliveries at any time. Throughout the Programme, participants have unlimited access to clinical support via secure messaging with physicians and pharmacists to discuss side effects, address concerns, or adjust therapy.

All data were extracted from the electronic health records database. Clinical data included the baseline pharmacological treatment prescribed, based on patient‐specific combinations of the following products: oral 5‐alpha‐reductase inhibitors (finasteride, dutasteride, or fixed‐dose combinations with minoxidil), oral minoxidil, topical minoxidil 5%, and adjuvant treatments (e.g., biotin, saw palmetto, anti‐hair loss shampoos). For analytical purposes, treatments were grouped according to their expected therapeutic potency into three categories: high potency (e.g., oral minoxidil and/or dutasteride, including combinations with topical agents), moderate potency (e.g., topical minoxidil or finasteride monotherapy), and low potency or cosmetic only (e.g., biotin, shampoo, or saw palmetto without pharmacological agents).

Treatments were categorized a priori according to pharmacological intensity and published evidence from trials, reviews and guideline summaries [8, 9, 10, 13, 16] Regimens containing oral dutasteride and/or oral minoxidil were classified as higher‐potency interventions because they included systemic agents with evidence of clinically relevant effects on hair growth. Oral finasteride and topical minoxidil 5% were classified as moderate‐potency treatments, reflecting their established first‐line role and well‐described efficacy. Non‐pharmacological or cosmetic‐only interventions were classified as low potency because evidence supporting clinically meaningful efficacy as standalone treatments is limited. This classification was intended as a pragmatic analytical framework rather than a definitive hierarchy of treatment effect.

Treatment classification was based on the baseline prescribed regimen. For participants receiving combination therapy, the initial combination was used for categorization. Adherence was assessed at the programme level using subscription renewal and cancelation data; granular adherence to individual treatment components could not be reliably quantified.

Quality of life was assessed using the Women's Androgenetic Alopecia Quality of Life Questionnaire, Brazilian Portuguese version (WAA‐QoL‐BP), a 16‐item questionnaire developed to evaluate the impact of alopecia on emotional, social, aesthetic, and functional aspects of wellbeing. Participants were asked to indicate the extent to which each statement reflected their experience over the past week. In the analyzed dataset, each item was scored from 1 to 7, giving a total score range of 16–112. Lower scores correspond to better quality of life, and any decrease in score over time reflects improvement. Although originally validated for use in women with androgenetic alopecia, the instrument was linguistically adapted for this study to improve relevance and comprehension among male patients, given the absence of a validated male‐specific QoL tool for alopecia in Brazil (see Supporting Information for the adapted questionnaire [17]).

#### 2.3.1 Psychometric Assessment of the Adapted Instrument

As the WAA‐QoL instrument was originally developed and validated in female populations, additional psychometric assessment was undertaken in the present male cohort. Internal consistency was evaluated using Cronbach's alpha. Construct validity was explored using confirmatory factor analysis (CFA) assuming a unidimensional structure and exploratory factor analysis (EFA) informed by Horn's parallel analysis.

Internal consistency was excellent (Cronbach's alpha = 0.96). Item–item correlations were generally moderate to high and item‐total correlations were high, supporting internal reliability in this cohort. A one‐factor CFA showed only modest fit, suggesting that a single underlying construct may not fully capture the structure of the questionnaire in men. Subsequent EFA suggested a possible three‐factor structure with improved fit. These analyses support internal reliability but do not constitute full validation of the instrument in male AGA populations.

The questionnaire was administered digitally. Upon purchase of any hair loss treatment product, participants automatically received an invitation to complete the baseline QoL assessment prior to their medical consultation. Subsequently, follow‐up assessments were scheduled to occur every three months, regardless of whether the participant attended additional consultations. At each follow‐up time point, a reminder message with a direct survey link was sent via an email. This process was fully automated and independent of patient login; participants were not required to access the treatment platform—they simply clicked the link to complete the questionnaire online.

Participants were invited to complete questionnaires monthly, with allowable variability of ±15 days around each nominal monthly time point. Responses outside this interval were assigned to the subsequent month. Accordingly, the six‐month follow‐up analysis reflects responses completed within approximately 5.5–6.5 months from baseline.

Adherence was defined using longitudinal renewal and cancelation data from the digital platform. Because actual medication intake could not be observed, “100% adherence” was operationalized as uninterrupted subscription renewals over the six‐month period, with no gaps or cancelations. Participants were classified as “< 100% adherence” if there was any lapse in their subscription, regardless of duration, but they later resumed treatment and completed the six‐month follow‐up. This metric reflects engagement with the treatment programme (i.e., continuing to purchase and receive products) rather than verified medication use.

### 2.4 Outcomes

The primary outcome was the change in quality of life (QoL) scores over a 6‐month period, assessed using the WAA‐QoL‐BP (Women's Androgenetic Alopecia Quality of Life, Brazilian Portuguese version) questionnaire [17]. Secondary outcomes of interest were: (1) changes to sub‐domains of the QoL score; (2) comparison of which sub‐domains were most affected by AGA treatment; and (3) changes in QoL scores by treatment adherence status, age, and medical treatment.

### 2.5 Ethical Considerations

The study protocol was reviewed and approved by the Local Research Ethics Committee, CAAE: 88067125.0.0000.5483. All procedures were conducted in accordance with the ethical standards of the responsible committee and with the principles of the Declaration of Helsinki (1975), as revised in 2000. Given the retrospective nature of the study, which used data routinely collected during clinical care, a waiver of informed consent was requested and granted. No written consent has been obtained from the patients as there is no patient‐identifiable data included. All data were anonymized prior to analysis to ensure participant confidentiality, and no identifiable information was accessed or disclosed at any point during the study (Resolutions CNS 466/12, 510/16, and the General Data Protection Law—LGPD).

### 2.6 Statistics

No formal sample size calculation was conducted, as this is a real‐world retrospective cohort study that included all eligible male users of the digital platform within the observation window. This pragmatic approach aimed to reflect routine care patterns and maximize generalizability. The change in QoL scores between baseline and six months was calculated (6‐month result minus baseline). The total QOL score was calculated for each patient by summing the responses to each of the 16‐hair loss‐related questions. Counts and percentages were used to summarize categorical data, and the mean and standard deviation for continuous variables. Univariate analysis of differences between the change in total QOL score by adherence and medicine potency groups was tested using Mann–Whitney and Kruskal–Wallis's hypothesis tests, respectively. A multivariable linear regression model was used to test for associations between the change in total QOL score and patient age, type of adherence, medical potency, and baseline QOL. Paired *t*‐tests were applied to compare the mean QOL scores at baseline vs. follow‐up both for total score and for each QOL question individually. A *p*‐value of < 0.05 was used as the threshold for statistical significance.

No formal correction for multiple testing was applied to analyses of individual QoL items. These analyses were considered exploratory and descriptive, and findings should therefore be interpreted cautiously, particularly for domains with smaller effect sizes.

## 3 Results

Out of 644 participants who completed the follow‐up questionnaire, 580 met eligibility criteria. The mean age at baseline was 31.80 years (SD 7.63). Of the analysis cohort, 376 (64.8%) were classified as 100% adherence throughout the six‐month period, 196 (33.8%) were classified as less than 100% adherent, and 8 had no adherence data (1.4%).

**FIGURE 1:**
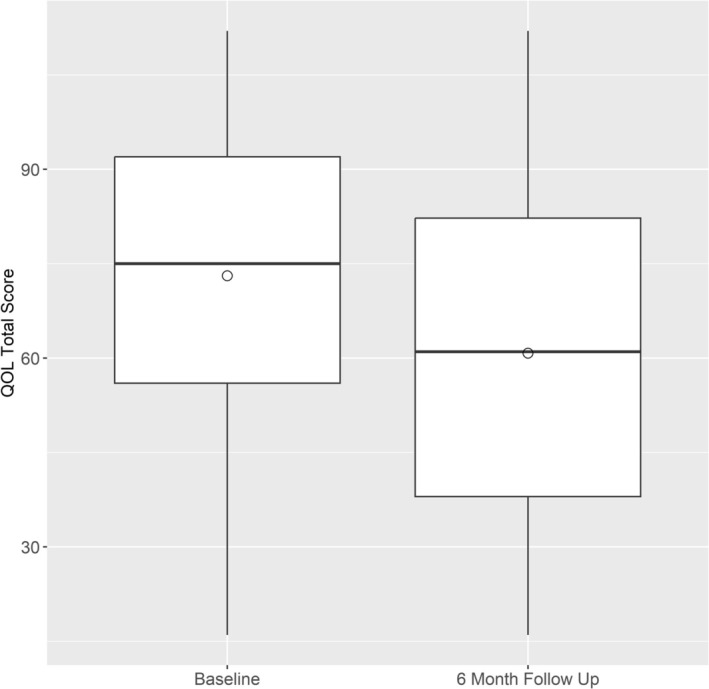
Box‐and‐whisker plot comparing mean and median QOL total score at baseline vs. 6 month follow up. The circle is the mean total QOL score. The horizontal line is the median, the box is the interquartile range, and the whiskers represent values above and below the upper and lower quartiles.

**FIGURE 2:**
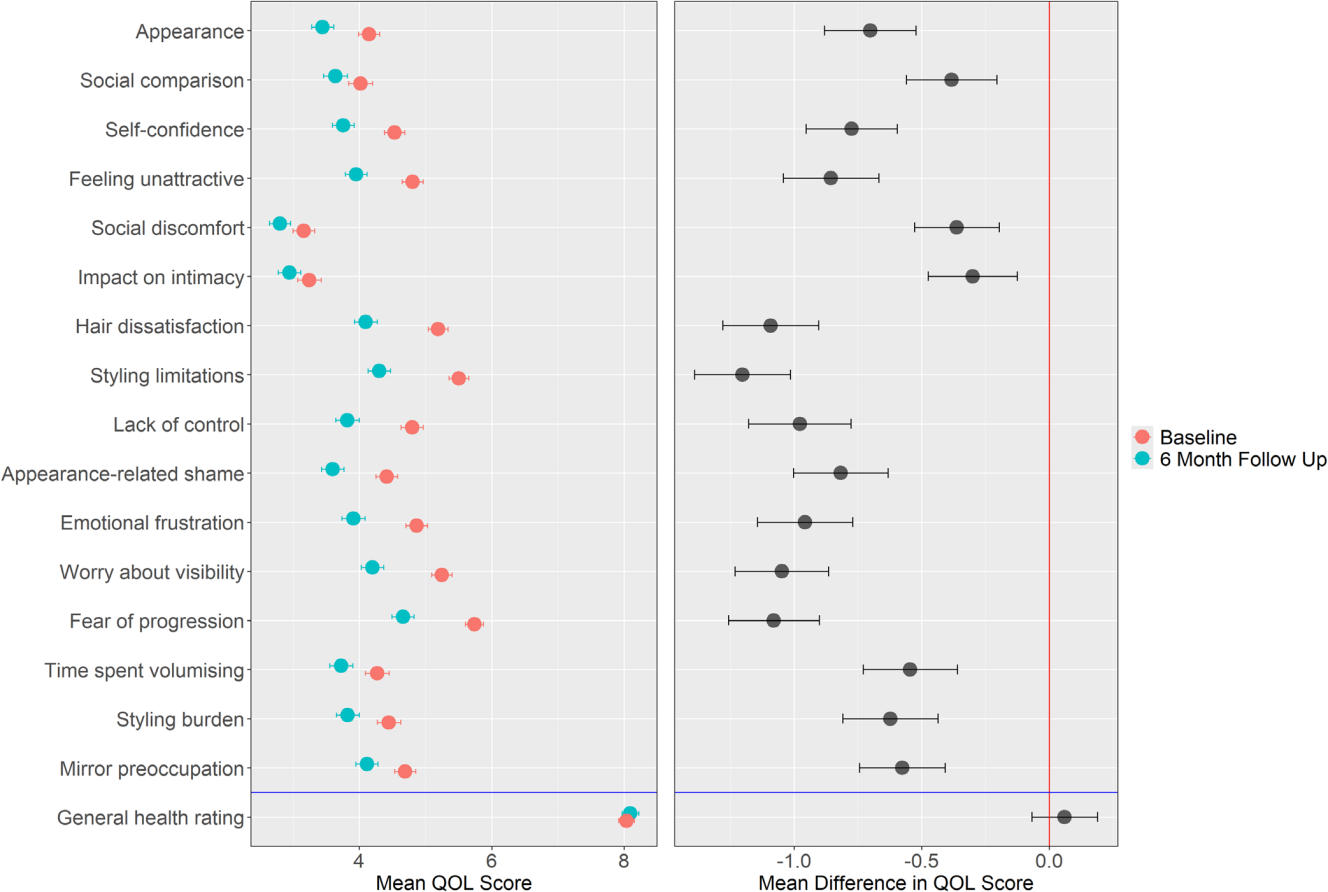
Left hand plot: The mean symptom score at baseline (red) and 6 month follow up (blue) together with their 95% confidence intervals. Right hand plot: The difference in symptom scores between baseline and 6 month follow up with 95% confidence intervals.

The mean QOL scores at baseline and 6‐month follow‐up for each QOL item are presented in Table 1. The mean total QOL score was 89.07 at baseline and 76.78 at 6‐month follow‐up (Table 1 and Figures 1 and 2). This reduction of 12.29 points (95% CI: −14.44 to −10.14; *p* < 0.001) represents an improvement in QoL because, on the WAA‐QoL‐BP, lower scores indicate better quality of life. There was no change from baseline to six months for general health rating (*p* = 0.359).

**TABLE 1:** The mean QOL scores with 95% confidence intervals for the baseline, 6 month follow up.

Questions	Total (*n* = 580)		100% Adherence (*n* = 376)		< 100% Adherence (*n* = 196)	
	Baseline	6‐month follow‐up	Baseline	6‐month follow‐up	Baseline	6‐month follow‐up
	Mean (95% CI)	Mean (95% CI)	Mean (95% CI)	Mean (95% CI)	Mean (95% CI)	Mean (95% CI)
General health	8.04 (7.92, 8.15)	8.10 (7.97, 8.22)	8.11 (7.98, 8.25)	8.21 (8.07, 8.35)	7.86 (7.65, 8.08)	7.86 (7.60, 8.12)
Q1 Appearance	5.15 (4.99, 5.31)	4.44 (4.28, 4.61)	5.08 (4.89, 5.28)	4.18 (3.98, 4.38)	5.22 (4.94, 5.51)	4.91 (4.63, 5.20)
Q2 Social comparison	5.02 (4.84, 5.20)	4.64 (4.46, 4.82)	4.87 (4.65, 5.09)	4.55 (4.33, 4.77)	5.24 (4.93, 5.56)	4.76 (4.44, 5.08)
Q3 Self‐confidence	5.53 (5.38, 5.69)	4.76 (4.59, 4.92)	5.48 (5.30, 5.67)	4.52 (4.32, 4.72)	5.60 (5.32, 5.87)	5.15 (4.87, 5.43)
Q4 Feeling unattractive	5.81 (5.65, 5.96)	4.95 (4.79, 5.11)	5.74 (5.54, 5.93)	4.73 (4.53, 4.93)	5.94 (5.66, 6.22)	5.33 (5.05, 5.61)
Q5 Social discomfort	4.16 (4.00, 4.33)	3.80 (3.64, 3.96)	4.02 (3.81, 4.22)	3.55 (3.35, 3.74)	4.39 (4.11, 4.68)	4.20 (3.92, 4.49)
Q6 Impact on intimacy	4.24 (4.07, 4.42)	3.94 (3.78, 4.11)	4.15 (3.93, 4.38)	3.69 (3.49, 3.89)	4.38 (4.08, 4.68)	4.35 (4.06, 4.64)
Q7 Hair dissatisfaction	6.19 (6.04, 6.34)	5.10 (4.93, 5.27)	6.24 (6.06, 6.43)	4.89 (4.68, 5.10)	6.07 (5.80, 6.34)	5.42 (5.13, 5.72)
Q8 Styling limitations	6.50 (6.36, 6.65)	5.30 (5.13, 5.47)	6.53 (6.35, 6.71)	5.08 (4.87, 5.29)	6.42 (6.15, 6.68)	5.67 (5.38, 5.97)
Q9 Lack of control	5.80 (5.63, 5.97)	4.82 (4.64, 5.00)	5.78 (5.57, 5.99)	4.53 (4.32, 4.75)	5.82 (5.54, 6.11)	5.30 (4.99, 5.61)
Q10 Appearance‐related shame	5.42 (5.25, 5.58)	4.60 (4.43, 4.77)	5.35 (5.15, 5.56)	4.33 (4.13, 4.54)	5.51 (5.23, 5.79)	5.04 (4.74, 5.34)
Q11 Emotional frustration	5.87 (5.70, 6.03)	4.91 (4.74, 5.08)	5.82 (5.61, 6.03)	4.66 (4.44, 4.87)	5.93 (5.67, 6.20)	5.34 (5.05, 5.63)
Q12 Worry about visibility	6.25 (6.09, 6.40)	5.20 (5.03, 5.37)	6.14 (5.94, 6.33)	5.00 (4.79, 5.21)	6.43 (6.17, 6.69)	5.54 (5.25, 5.83)
Q13 Fear of progression	6.74 (6.60, 6.87)	5.66 (5.49, 5.83)	6.73 (6.56, 6.90)	5.47 (5.26, 5.68)	6.72 (6.48, 6.97)	6.02 (5.74, 6.29)
Q14 Time spent volumising	5.27 (5.09, 5.45)	4.73 (4.55, 4.90)	5.12 (4.90, 5.34)	4.46 (4.25, 4.67)	5.51 (5.21, 5.82)	5.17 (4.88, 5.47)
Q15 Styling burden	5.45 (5.27, 5.62)	4.82 (4.65, 5.00)	5.38 (5.16, 5.61)	4.56 (4.35, 4.77)	5.53 (5.23, 5.82)	5.26 (4.97, 5.56)
Q16 Mirror preoccupation	5.69 (5.53, 5.85)	5.11 (4.95, 5.28)	5.64 (5.45, 5.84)	4.84 (4.63, 5.04)	5.75 (5.47, 6.03)	5.59 (5.31, 5.87)
**Total score**	**89.07 (87.08, 91.06)**	**76.78 (74.51, 79.06)**	**88.09 (85.64, 90.53)**	**73.04 (70.25, 75.82)**	**90.47 (86.97, 93.97)**	**83.06 (79.21, 86.91)**

*Note:* Each WAA‐QoL‐BP item was scored from 1 to 7; the total score is the sum of the 16 hair‐loss items (range 16–112). Lower scores indicate better quality of life. General health is rated on a separate scale and is not included in the total. For full descriptions of each item, please see the Supporting Information.

**FIGURE 3:**
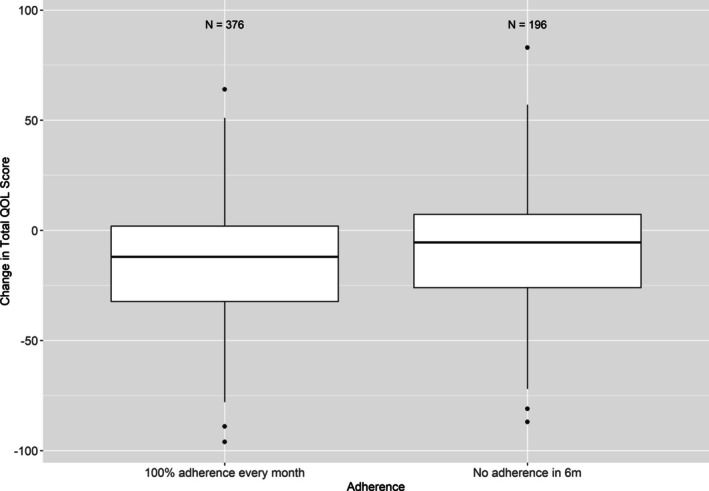
Box‐and‐whisker plot comparing mean and median change in QOL total score by adherence type. The circle is the mean total QOL score. The horizontal line is the median; the box is the interquartile range, the whiskers represent values above and below the upper and lower quartiles, and the individual dots are outliers.

When the data were stratified by adherence, the baseline mean score among those with less than 100% adherence showed a mean improvement of 7.41 points (95% CI −11.45, −3.37) compared to those with 100% adherence who showed a mean improvement of 15.05 points (95% CI −17.57, −12.53) (Table 1, Figure 3).

The coefficients of the multivariable regression model are provided in Table 2. The change in total QOL score was found to be associated with the baseline total QOL score (*p*‐value < 0.001), adherence (*p*‐value < 0.001), and age (*p*‐value = 0.024), but not medication potency (*p*‐value = 0.119) (Table 3).

**TABLE 2:** Results of the multivariable linear regression of the change in total QOL score between baseline and 6 month follow up.

	Coefficient	Standard error	95% CI	*T* statistic	*p*
(Intercept)	26.305	5.842	(14.83, 37.78)	4.502	< 0.001
Baseline total QOL score	−0.465	0.042	(−0.548, −0.383)	−11.115	< 0.001
Age	−0.273	0.133	(−0.534, −0.012)	−2.054	0.040
Adherence: 100% adherence every month	−8.813	2.108	(−12.953, −4.672)	4.180	< 0.001
Medication group: moderate potency	11.904	7.258	(−2.352, 26.16)	1.640	0.102
Medication group: low potency	5.734	4.288	(−2.688, 14.156)	1.337	0.182

Abbreviation: QOL, quality of life.

**TABLE 3:** The analysis of variance for the multivariable linear regression of the change in total QOL score between baseline and 6 month follow up.

	Df	Sum sq	Mean sq	*F*	*p*
Baseline total QOL score	1	65.431	65 431	115.955	< 0.001
Age	1	2882	2882	5.108	0.024
Adherence	1	9489	9489	16.816	< 0.001
Medication group	3	3.319	1106	1.961	0.119

Abbreviations: df, degrees of freedom; *F*‐value, test statistic from ANOVA; Mean Sq, mean square; QOL, quality of life; Sum Sq, sum of squares.

The results highlight that, on average, the worse the total QOL at baseline, the greater the improvement in total QOL symptoms at 6‐month follow‐up. The total QOL score improved on average 0.273 points for every year older a man was (95% CI −0.534, −0.012).

Out of the 16 questions, the mean change in QoL score was greater than 1 point on the Likert scale for four questions (the remaining questions changed by less than 1 point on average) (Table 4). The four questions showing the largest improvements from baseline related to the emotional and behavioral impact of hair loss. The items with the greatest average improvement over six months were predominantly related to emotional distress, behavioral coping mechanisms, and anticipatory concerns.

**TABLE 4:** Survey items with the most significant change at 6 months of follow‐up.

Question	Description	Mean change	*p*
Q8	Feeling helpless about hair loss	−1.20	< 0.001
Q7	Hair loss affected hair styling	−1.09	< 0.001
Q12	Concern about continued hair loss	−1.05	< 0.001
Q13	Time spent trying to make hair look fuller	−1.08	< 0.001

The largest mean improvements over six months were observed for feeling helpless about hair loss (−1.20), hair styling limitations (−1.09), time spent making hair look fuller (−1.08), and concern about continued hair loss (−1.05). These changes each exceeded one point on a 7‐point Likert scale and may therefore be more perceptible to patients in everyday life. However, because item‐level analyses were exploratory and not adjusted for multiple testing, these findings should be interpreted as descriptive patterns rather than confirmatory evidence of domain‐specific treatment effects.

## 4 Discussion

This study offers real‐world evidence that sustained engagement with a fully digital care programme for androgenetic alopecia can lead to clinically meaningful improvements in hair‐loss‐related quality of life over six months. In this context, “adherence” referred to uninterrupted participation in the Programme—continuous prescription renewals and interaction with the platform—rather than confirmed ingestion of the prescribed products. Thus, the observed benefits likely reflect both the pharmacologic effect and the ongoing support provided by the digital service, including remote consultations, follow‐ups and home delivery of medications. Significant improvements were recorded overall and across multiple QoL domains, with larger gains among men who started with poorer QoL, those who maintained programme adherence and older participants. The greatest improvements occurred in domains related to emotional distress, behavioral coping and anticipatory concerns about hair loss, underscoring the ability of digital care models to alleviate both the psychological and practical burdens of AGA.

Over the six‐month period, the greatest improvements in QoL clustered around themes of emotional distress, behavioral coping, and anticipatory concerns. Feelings of helplessness (Q9) and shame (Q10) declined by nearly a point, suggesting that the programme offers more than pharmacologic benefit. Its features—including asynchronous consultations, educational resources on treatment timelines and side effects, and a progress‐tracking tool for uploading photos and receiving personalized feedback—likely helped alleviate these emotional burdens.[14] In the domain of behavioral coping, substantial reductions in time spent making hair look fuller (Q14) and difficulty styling hair (Q8) point to tangible relief in daily routines, perhaps fostered by clear expectations and ongoing support. Similarly, improved scores for concerns about continued hair loss (Q13) and visibility of bald spots (Q12) imply that continuous engagement with the programme helps restore a sense of control. Taken together, these patterns indicate that sustained participation in a fully digital care pathway can ease both the psychological and practical consequences of androgenetic alopecia.

Across all 16 hair‐loss–specific items, scores improved significantly over six months, while the general health rating remained stable, serving as a useful “negative control.” The four largest mean reductions—helplessness, styling limitations, concern about continued hair loss, and time spent making hair look fuller—ranged from 1.05 to 1.20 points. On a seven‐point Likert scale, such changes are generally considered clinically meaningful [18]. In contrast, some items had smaller yet statistically significant decreases that may not be perceptible in daily life. This distinction highlights the value of focusing on domains where improvements surpass clinically relevant thresholds and supports the potential benefits of the digital care model.

Not all QoL domains improved to the same extent. Domains relating to social interaction, intimacy, and appearance‐related self‐consciousness appeared to change more modestly. This may reflect the time required for visible hair regrowth to translate into psychosocial benefit, as well as the possibility that some psychological effects of hair loss are longstanding and less readily reversible over a short follow‐up period.

Androgenetic alopecia (AGA) is not only a cosmetic issue but also a condition with substantial psychological burden. In a systematic review and meta‐analysis, Huang et al. [3] showed that AGA impairs quality of life (QoL), with 41 studies and 7995 patients. The pooled DLQI score was 8.16 (95% CI, 5.62–10.71), and the Hair‐Specific Skindex‐29 emotion subscale was 29.22 (95% CI, 24.17–34.28), both reflecting moderate impacts.

In our study, programme adherence—defined as continuous engagement with the digital healthcare platform—was a key determinant of QoL improvement. This reflects commitment to care rather than medication intake per se. This finding aligns with Nestor et al. [8], who noted that long‐term adherence is essential for AGA treatment success and that decisions should account for patient preferences and practical constraints. Our results suggest that the Programme's features—such as asynchronous consultations, educational content, and ongoing support—may enhance engagement and yield meaningful QoL gains.

The < 100% adherence group was heterogeneous and likely included both brief interruptions and more prolonged disengagement. This may have attenuated the observed association between adherence and QoL improvement. Future studies should prospectively define adherence categories with greater granularity, including duration and timing of treatment interruptions.

The association between lower baseline QoL and greater subsequent improvement should also be interpreted with caution. This pattern may partly reflect regression to the mean, whereby individuals with more extreme baseline scores tend to show greater change over time independent of treatment effect.

Notably, medication potency or type was not independently associated with QoL improvement. This supports findings by Liborio et al. [19], who observed no significant differences between minoxidil alone and its combination with bicalutamide in women. However, small sample sizes in our subgroups (*n* = 11, 33, and 33) limited statistical power. Large standard errors in Table 2 reflect this uncertainty. More data would allow better evaluation of medication effects on QoL. These findings suggest that patient‐related factors—expectations, motivation, and consistency—may outweigh pharmacologic differences, particularly for subjective outcomes.

Most prior AGA studies focus on clinical endpoints like hair density, with fewer addressing patient‐reported outcomes [14, 15]. Studies by Sinclair and Biondo (2010), Zhuang et al. (2013), Singh et al. (2023), and Krefft‐Trzciniecka et al. (2024) emphasized QoL improvements and psychosocial factors in women with alopecia [16, 20, 21]. Our study adds to this literature by documenting longitudinal QoL improvements in men treated via a digital platform.

The digital setting adds an important perspective. Unlike studies in clinical environments, we examined a real‐world population using digital care. A 12‐month RCT in psoriasis patients showed that collaborative online care yielded QoL gains comparable to in‐person care, with DLQI and Skindex‐16 scores within equivalence margins [22]. These findings support digital platforms as viable tools for chronic dermatologic care.

The digital care model also introduces potential limitations. Diagnostic assessment is performed without in‐person examination, which may increase the risk of misclassification in a minority of cases. Prescribing is protocol‐driven and may not capture all clinical nuances. Long‐term engagement may vary, and follow‐up data may be incomplete. These factors should be considered when interpreting real‐world outcomes from digital platforms.

A key strength of this study is its use of real‐world data from a Brazilian digital healthcare platform, enabling assessment of quality‐of‐life outcomes and programme engagement in 580 men treated entirely online. These platforms offer asynchronous consultations, automated treatment initiation, educational materials, progress‐tracking tools and home delivery of medications, aiming to reduce logistical barriers and improve engagement [14]. To our knowledge, this is among the first studies to evaluate such a model for AGA in Brazil, and the large sample size enhances the statistical power. Including the general health rating as a “negative control”—an item not expected to change with hair‐loss treatment—helped reduce bias: this score remained stable while hair‐loss‐specific domains improved, supporting the programme's real‐world effectiveness.

We acknowledge the study's limitations. First, the WAA‐QoL‐BP instrument was originally validated for women and was linguistically adapted for this male cohort due to the lack of a male‐specific tool in Brazil. Although internal consistency was high in this sample, the additional psychometric analyses do not amount to full validation in male AGA populations. Second, the retrospective observational design limits causal inference, and improvements in QoL cannot be attributed solely to treatment effects; they may reflect a combination of treatment, engagement with care, expectation effects, and natural variation over time. Third, psychiatric comorbidities and major life events, which may influence QoL independently of AGA, were not systematically captured and could not be included in the analysis. Residual confounding therefore remains possible. Fourth, the six‐month follow‐up may not capture longer‐term QoL changes or adherence trends. Fifth, our adherence metric reflects ongoing programme participation, defined by prescription renewals and cancelation data, rather than confirmed medication intake; therefore, the < 100% adherence group is heterogeneous. Lastly, some patient groups, such as those with missing adherence data or without six‐month follow‐up, were excluded due to small sample sizes.

Future studies would benefit from incorporating direct patient‐reported assessments of perceived hair regrowth or clinical improvement alongside validated QoL measures. While QoL captures the psychosocial impact of treatment, parallel assessment of perceived efficacy may help better contextualize changes in patient experience and treatment satisfaction.

Although the observed improvements in QoL were statistically significant, their clinical meaningfulness warrants careful interpretation. The magnitude of change should be considered in the context of baseline scores, instrument properties, and the absence of an established minimal clinically important difference for this adapted measure in male populations.

## 5 Conclusion

This retrospective cohort study found statistically significant improvements in hair‐loss‐related quality of life over six months among men treated for androgenetic alopecia through a fully digital programme. Improvements were larger among participants with continuous subscription renewals, suggesting that sustained engagement with the programme may be an important marker of better patient‐reported outcomes. The largest changes related to emotional distress, behavioral burden, and anticipatory concerns about ongoing hair loss. These findings support the potential value of digital platforms in delivering patient‐centered AGA care, but they should be interpreted in light of the observational design, absence of an in‐person comparator, residual confounding, and the need for full psychometric validation of the adapted questionnaire in male populations.

## Author Contributions

Gabriel Guimarães and Angela C. Bersch‐Ferreira conceived the study and developed the study design protocol. Angela C. Bersch‐Ferreira and John C.T. Chao were responsible for data acquisition. Daniel Reisel, Hans Johnson, and Ashley K. Clift conducted the data analysis and statistical analysis. Angela C. Bersch‐Ferreira, Daniel Reisel, Hans Johnson, and Ashley K. Clift drafted the first version of the manuscript. David R. Huang and Gabriel Guimarães provided supervision, contributed to the interpretation of the results, and critically revised the manuscript for important intellectual content. Hudson Dutra contributed to the interpretation of the results and critically revised the manuscript for important intellectual content. Gabriel Guimarães is the guarantor of the work.

## Funding

This study was not supported by any external sponsor or funder. MANUAL provided access to anonymized data for research purposes.

## Ethics Statement

This study protocol was reviewed and approved by Beneficência Portuguesa Hospital Ethics Committee, from São Paulo‐Brazil (approval number CAAE: 88067125.0.0000.5483).

## Consent

Written informed consent was waived for this study, as it involved retrospective analysis of routinely collected data from a digital healthcare platform. All users had previously agreed, at the time of enrolment in the programme, to the use of their anonymised data for research purposes, in accordance with the platform's terms of use and privacy policy. The waiver of informed consent was approved by the local Research Ethics Committee, which determined that the study posed minimal risk to participants and that obtaining individual consent was impracticable given the retrospective nature of the data and the large sample size.

## Conflicts of Interest

A.C.B.‐F., J.C.T.C., and G.G. are employed by MANUAL LTDA, and D.R., A.K.C., H.J., and D.R.H. are employed by Menwell Limited, the sponsoring company for this study.

## Supporting information


**Data S1:** The completed STROBE checklist for observational studies and the linguistically adapted version of the WAA‐QoL‐BP questionnaire used in this study.

## Data Availability

The datasets generated and/or analyzed during the current study are not publicly available due to legal and privacy constraints, as they are derived from a private digital healthcare platform. However, de‐identified data may be made available from the corresponding author upon reasonable request and with permission from MANUAL.
