# Supplementary material for: Quality of Life in Men Treated for Androgenetic Alopecia via a Digital Healthcare Platform: A Retrospective Cohort Study
Source: J Cosmet Dermatol. 2026 Jun 17;25(6):e70999. doi: 10.1111/jocd.70999 (PMC13276016; doi:10.1111/jocd.70999)
Supplement: Supplementary file 1 — Data S1: The completed STROBE checklist for observational studies and the linguistically adapted version of the WAA‐QoL‐BP questionnaire used in this study. [file JOCD-25-e70999-s001.docx]

##### **Research Article** **Quality of life in men treated for androgenetic alopecia via a digital healthcare platform: a retrospective cohort study**

**Supplementary Material**

**STROBE Statement—Checklist of items that should be included in reports of *cohort studies***

|  | **Item No** | **Recommendation** | **Page** |
| --- | --- | --- | --- |
| **Title and abstract** | 1 | (*a*) Indicate the study’s design with a commonly used term in the title or the abstract | 1 |
|  |  | (*b*) Provide in the abstract an informative and balanced summary of what was done and what was found | 1 |
| **Introduction** | | |  |
| Background/rationale | 2 | Explain the scientific background and rationale for the investigation being reported | 2-3 |
| Objectives | 3 | State specific objectives, including any prespecified hypotheses | 3 |
| **Methods** | | |  |
| Study design | 4 | Present key elements of study design early in the paper | 3 |
| Setting | 5 | Describe the setting, locations, and relevant dates, including periods of recruitment, exposure, follow-up, and data collection | 3 |
| Participants | 6 | (*a*) Give the eligibility criteria, and the sources and methods of selection of participants. Describe methods of follow-up | 4 |
|  |  | (*b*) For matched studies, give matching criteria and number of exposed and unexposed | - |
| Variables | 7 | Clearly define all outcomes, exposures, predictors, potential confounders, and effect modifiers. Give diagnostic criteria, if applicable | 5-6 |
| Data sources/ measurement | 8* | For each variable of interest, give sources of data and details of methods of assessment (measurement). Describe comparability of assessment methods if there is more than one group | *4-5* |
| Bias | 9 | Describe any efforts to address potential sources of bias | 5 |
| Study size | 10 | Explain how the study size was arrived at | 7 |
| Quantitative variables | 11 | Explain how quantitative variables were handled in the analyses. If applicable, describe which groupings were chosen and why | 6 |
| Statistical methods | 12 | (*a*) Describe all statistical methods, including those used to control for confounding | 7 |
|  |  | (*b*) Describe any methods used to examine subgroups and interactions | 7 |
|  |  | (*c*) Explain how missing data were addressed |  |
|  |  | (*d*) If applicable, explain how loss to follow-up was addressed | - |
|  |  | (*e*) Describe any sensitivity analyses | - |
| **Results** | | |  |
| Participants | 13* | (a) Report numbers of individuals at each stage of study—eg numbers potentially eligible, examined for eligibility, confirmed eligible, included in the study, completing follow-up, and analysed | 8 |
|  |  | (b) Give reasons for non-participation at each stage | 8 |
|  |  | (c) Consider use of a flow diagram | - |
| Descriptive data | 14* | (a) Give characteristics of study participants (eg demographic, clinical, social) and information on exposures and potential confounders | 8 |
|  |  | (b) Indicate number of participants with missing data for each variable of interest | Table |
|  |  | (c) Summarise follow-up time (eg, average and total amount) | 8-9 |
| Outcome data | 15* | Report numbers of outcome events or summary measures over time | 8-9 |
| Main results | 16 | (*a*) Give unadjusted estimates and, if applicable, confounder-adjusted estimates and their precision (eg, 95% confidence interval). Make clear which confounders were adjusted for and why they were included | 8-9 |
|  |  | (*b*) Report category boundaries when continuous variables were categorized | 8-9 |
|  |  | (*c*) If relevant, consider translating estimates of relative risk into absolute risk for a meaningful time period | - |
| Other analyses | 17 | Report other analyses done—eg analyses of subgroups and interactions, and sensitivity analyses | 8-9 |
| **Discussion** | | |  |
| Key results | 18 | Summarise key results with reference to study objectives | 9-10 |
| Limitations | 19 | Discuss limitations of the study, taking into account sources of potential bias or imprecision. Discuss both direction and magnitude of any potential bias | 10 |
| Interpretation | 20 | Give a cautious overall interpretation of results considering objectives, limitations, multiplicity of analyses, results from similar studies, and other relevant evidence | 10 |
| Generalisability | 21 | Discuss the generalisability (external validity) of the study results | 10-12 |
| **Other information** | | |  |
| Funding | 22 | Give the source of funding and the role of the funders for the present study and, if applicable, for the original study on which the present article is based | Cover Letter |

**Table 1S.** Description of the Questionnaire Items

| **Question ID** | **Description** |
| --- | --- |
| QoL General | How would you rate your general health today?  [1 – The worst you can imagine, 10 – The best you can imagine] |
| QoL Q1 | In the past WEEK, how self-conscious did you feel because people looked at your hair?  ⎕ Extremely ⎕ Very much ⎕ Quite a lot ⎕ Somewhat ⎕ A little ⎕ Very little ⎕ Not at all |
| QoL Q2 | In the past WEEK, how much did you envy / feel jealous of people who have a lot of hair?  ⎕ Extremely ⎕ Very much ⎕ Quite a lot ⎕ Somewhat ⎕ A little ⎕ Very little ⎕ Not at all |
| QoL Q3 | In the past WEEK, how much did your hair loss NEGATIVELY affect your self-confidence?  ⎕ Extremely ⎕ Very much ⎕ Quite a lot ⎕ Somewhat ⎕ A little ⎕ Very little ⎕ Not at all |
| QoL Q4 | In the past WEEK, how unattractive did you feel because of your hair loss?  ⎕ Extremely ⎕ Very much ⎕ Quite a lot ⎕ Somewhat ⎕ A little ⎕ Very little ⎕ Not at all |
| QoL Q5 | In the past WEEK, how problematic was it for you to socialize with people you don’t know because of your hair loss?  ⎕ Extremely ⎕ Very much ⎕ Quite a lot ⎕ Somewhat ⎕ A little ⎕ Very little ⎕ Not at all |
| QoL Q6 | In the past WEEK, how problematic was it for you to interact with people of the opposite sex (or same sex) because of your hair loss?  ⎕ Extremely ⎕ Very much ⎕ Quite a lot ⎕ Somewhat ⎕ A little ⎕ Very little ⎕ Not at all |
| QoL Q7 | In the past WEEK, how much did your hair loss NEGATIVELY affect your satisfaction with how your hair looks?  ⎕ Extremely ⎕ Very much ⎕ Quite a lot ⎕ Somewhat ⎕ A little ⎕ Very little ⎕ Not at all |
| QoL Q8 | In the past WEEK, how much did your hair loss NEGATIVELY affect the way you like to style your hair?  ⎕ Extremely ⎕ Very much ⎕ Quite a lot ⎕ Somewhat ⎕ A little ⎕ Very little ⎕ Not at all |
| QoL Q9 | In the past WEEK, how helpless (out of control) did you feel about doing something regarding your hair loss?  ⎕ Extremely ⎕ Very much ⎕ Quite a lot ⎕ Somewhat ⎕ A little ⎕ Very little ⎕ Not at all |
| QoL Q10 | In the past WEEK, how ashamed did you feel because of the appearance of your hair?  ⎕ Extremely ⎕ Very much ⎕ Quite a lot ⎕ Somewhat ⎕ A little ⎕ Very little ⎕ Not at all |
| QoL Q11 | In the past WEEK, how frustrated did you feel because of your hair loss?  ⎕ Extremely ⎕ Very much ⎕ Quite a lot ⎕ Somewhat ⎕ A little ⎕ Very little ⎕ Not at all |
| QoL Q12 | In the past WEEK, how worried were you about parting your hair and revealing areas (bald spots) on your scalp?  ⎕ Extremely ⎕ Very much ⎕ Quite a lot ⎕ Somewhat ⎕ A little ⎕ Very little ⎕ Not at all |
| QoL Q13 | In the past WEEK, how concerned were you that your hair loss might continue?  ⎕ Extremely ⎕ Very much ⎕ Quite a lot ⎕ Somewhat ⎕ A little ⎕ Very little ⎕ Not at all |
| QoL Q14 | In the past WEEK, how much time did you spend trying to make your hair look fuller and thicker because of hair loss?  ⎕ Extremely ⎕ Very much ⎕ Quite a lot ⎕ Moderate ⎕ A little ⎕ Very little ⎕ None |
| QoL Q15 | In the past WEEK, how bothered were you by the time spent fixing your hair to cover your scalp (bald spots) because of hair loss?  ⎕ Extremely ⎕ Very much ⎕ Quite a lot ⎕ Somewhat ⎕ A little ⎕ Very little ⎕ Not at all |
| QoL Q16 | In the past WEEK, how much time did you spend checking your hair in the mirror because of hair loss?  ⎕ Extremely ⎕ Very much ⎕ Quite a lot ⎕ Moderate ⎕ A little ⎕ Very little ⎕ None |

Legend: Qol, Quality of life; Q, Question
